# Physical Symptoms and Neurocognitive Complaints in Long COVID: Associations with Gender, Age, Education, and Clinical Factors

**DOI:** 10.3390/brainsci15111180

**Published:** 2025-10-30

**Authors:** Somayeh Pour Mohammadi, Razieh Etesamipour, Francisco Mercado Romero, Moein Noroozi Fashkhami, Irene Peláez

**Affiliations:** 1 Department of Psychology, School of Health Sciences, Rey Juan Carlos University, 28922 Madrid, Spain; s.pour.2020@alumnos.urjc.es (S.P.M.);; 2 Department of Psychology, Payame Noor University (PNU), Tehran 19395-4697, Iran; r_etesamipour@pnu.ac.ir; 3 Department of Clinical Psychology and Education, Central Tehran Branch, Islamic Azad University, Tehran 1955847781, Iran

**Keywords:** long COVID, neurocognitive complaints, physical symptoms, memory, attention, gender, age, education

## Abstract

Long COVID is frequently accompanied by neurocognitive complaints, yet the combined effects of demographic and clinical factors remain unclear. This study examined individuals six months after their most recent SARS-CoV-2 infection using a Demographic/Infection-History form, a Physical and Neurocognitive Symptom Checklist (binary), and the Post-COVID Cognitive Impairment Scale (memory, attention; 5-point Likert). Participants were recruited through convenience sampling from multiple community and online sources. Inclusion criteria required confirmed prior COVID-19 infection, self-perceived or clinically documented Long COVID symptoms, and no history of neurological or severe psychiatric disorders. The final sample consisted of 212 participants (mean age = 39.7 years, SD = 10.5), of whom 67.9% were female, and most held a master’s (35.4%) or bachelor’s (28.3%) degree. Difficulties with retaining new information (57.8%) and concentrating (52.1%) were the most frequent neurocognitive complaints, while severe fatigue after mild activity (23.2%) and chronic fatigue (22.7%) were the most common physical symptoms. Confusion and decision-making difficulty were more frequent among younger participants; women reported greater difficulty retaining new information, and difficulty concentrating varied by education level. A multivariable regression model explained 7% of the variance in total cognitive complaints, identifying education level (β = −0.18, *p* < 0.01) and number of physical symptoms (β = 0.19, *p* < 0.01) as significant predictors. Higher educational attainment was associated with fewer cognitive complaints, whereas a greater burden of physical symptoms predicted higher complaint scores. Persistent cognitive difficulties in Long COVID appear closely related to physical symptom burden and protective factors such as education, rather than to infection frequency or sensory dysfunction duration. Findings highlight the need for routine cognitive screening, fatigue-focused management, and longitudinal multimodal research to elucidate underlying mechanisms and recovery pathways.

## 1. Introduction

COVID-19, caused by SARS-CoV-2, has revealed itself as more than a respiratory illness, with mounting evidence pointing to enduring neurological and cognitive impairments [1]. Among the most concerning sequelae is Long COVID, a condition characterized by symptoms persisting beyond four weeks post-infection, affecting an estimated 10–30% of survivors [2,3]. This chronic phase often manifests as neurological and cognitive impairments, collectively termed “brain fog,” encompassing deficits in memory, attention, and processing speed [2,4]. Research suggests these impairments may be linked to mechanisms such as neuroinflammation, hypoxic brain injury, and microvascular damage [5], consistent with possible disruptdisruption of neural integrity [6]. Studies like those by Taquet et al. (2021) [1] and Davis et al. (2021) [2] have documented a broad spectrum of persistent symptoms—fatigue, shortness of breath, headache, and cognitive difficulties—highlighting their interplay with physical health, infection history (e.g., number of infections, sensory dysfunction), and demographic factors (e.g., gender, age, education) in shaping Long COVID outcomes [1,2,4]. Understanding how these variables relate to cognitive outcomes, particularly at six months post-infection, is therefore important for informing rehabilitation strategies.

### 1.1. Demographic Influences: Gender, Age, and Education

Demographic variables such as gender, age, and education are increasingly recognized as potential modulators of cognitive impairment in Long COVID [7]. Evidence suggests that while no gender differences were typically observed during the acute phase of infection, such disparities have emerged in the post-acute and chronic stages. For instance, Michelutti et al. [8] and Toepffer et al. [9], reported that women were more likely to experience persistent cognitive complaints—including concentration difficulties, fatigue, and “brain fog”—months after recovery, possibly reflecting sex-specific neuroinflammatory and hormonal responses [10,11]. Conversely, Ferrucci et al. [12] found no significant gender-related differences during hospitalization, emphasizing that acute-phase data may not capture the long-term cognitive impact characteristic of Long COVID.

Age has also been identified as a major determinant of cognitive outcomes. Zhou et al. [13] and Shah et al. [14] observed that older adults exhibited greater deficits in memory and processing speed, suggesting an interaction between viral neuroinflammation and age-related vulnerability [15]. However, longitudinal data indicate that these deficits are not solely age-dependent but may represent an accelerated cognitive aging trajectory triggered by SARS-CoV-2 infection [14].

The role of education remains comparatively underexplored, though recent evidence supports its protective function. Individuals with higher educational attainment tend to report fewer subjective cognitive complaints and perform better on objective neuropsychological measures, likely due to greater cognitive reserve and neural compensatory capacity [14]. These findings reinforce the notion that education may buffer against cognitive decline through enhanced neural plasticity and adaptive network efficiency [16,17].

Taken together, the fragmented evidence across demographic domains underscores the need for integrative research examining how gender, age, and education jointly modulate neurocognitive and physical symptom profiles in Long COVID [18].

### 1.2. Physical Symptom Profile and Cognitive Outcomes

Research has increasingly focused on how physical symptoms experienced during and after COVID-19 influence cognitive functioning among individuals with Long COVID [19]. Earlier studies demonstrated that non-hospitalized patients with persistent fatigue and dyspnea often exhibited deficits in attention and memory several months post-infection, although such studies lacked detailed examination of how specific symptom clusters relate to cognitive outcomes [20,21,22]. More recent evidence provides a broader perspective: meta-analytic and scoping reviews have confirmed that fatigue and cognitive impairment are among the most frequent and long-lasting manifestations of Long COVID [23,24]. These findings, together with more recent clinical observations linking post-exertional malaise and muscle abnormalities to neurocognitive dysfunction [25,26], underscore the need to systematically evaluate how the physical symptom profile is associated with subjective cognitive complaints in Long COVID.

### 1.3. Impact of Infection History: Number of Infections and Olfactory/Gustatory Dysfunction

The history of COVID-19 infections—both in terms of the number of episodes and the occurrence of sensory dysfunctions such as olfactory and gustatory loss—may influence cognitive outcomes in Long COVID, yet these factors remain insufficiently explored [27]. Repeated SARS-CoV-2 infections could produce cumulative inflammatory and vascular insults that adversely affect brain integrity, as indicated by studies linking greater symptom burden to poorer cognitive performance [4,28]. Although most investigations have not differentiated between single and multiple infections, emerging evidence suggests that repeated viral and immune activation may heighten the risk of persistent neurocognitive symptoms [29,30].

Olfactory and gustatory dysfunctions have been widely recognized as early and often co-occurring neurological manifestations of COVID-19. Both are believed to arise from overlapping peripheral and central neural pathways—including the olfactory bulb, orbitofrontal cortex, and temporal regions—implying a shared neuroinflammatory mechanism [26,29]. Empirical studies further indicate that patients seldom report one dysfunction without the other, and self-reported data frequently conflate them, supporting their combined consideration under the term olfactory/gustatory dysfunction [26,31]. From a neuropsychological perspective, these sensory alterations may serve as accessible clinical markers of neural involvement in Long COVID. For instance, Almeria et al. [31], found that individuals presenting anosmia and dysgeusia displayed memory impairment six months post-infection, suggesting a link between sensory dysfunction and cognitive decline [16,32]. Recent neuroimaging work further supports this association by demonstrating that olfactory deficits correlate with microstructural brain alterations related to cognitive impairment and fatigue [29]. Longitudinal studies also indicate that olfactory dysfunction may persist for years and parallel subtle cognitive decline [33]. While gustatory dysfunction often co-occurs, some evidence shows it may recover faster than olfactory impairment [34], reinforcing the pragmatic use of a combined sensory measure in self-report and clinical research.

Altogether, these findings suggest that both the number of infections and olfactory/gustatory dysfunction represent complementary pathways of neurological involvement that may contribute to persistent cognitive complaints in Long COVID [35].

### 1.4. Research Gaps and the Six-Month Post-Infection Focus

Given the chronic nature of Long COVID, understanding how the physical symptom profile, infection history, and demographic factors contribute to cognitive outcomes six months post-infection is critical for informing clinical management and rehabilitation strategies [36]. This timeframe holds particular significance because research, such as that by Taquet et al. [1], demonstrates that neurological and cognitive symptoms often persist beyond the acute phase, with a notable proportion of Long COVID patients showing persistent or emerging deficits around six months, marking a transition from subacute to chronic symptomology [1,37]. For instance, Davis et al. [2] found that over 85% of Long COVID patients report “brain fog” at this stage, with 70% experiencing persistent memory and attention deficits that impair daily activities like managing household tasks or maintaining social relationships, aligning with the World Health Organization’s emphasis on Long COVID’s prolonged effect on quality of life [2,38]. Furthermore, Hampshire et al. [4] highlighted that these cognitive impairments significantly disrupt work performance, particularly in roles requiring sustained attention (e.g., problem-solving, decision-making), with poorer cognitive test scores linked to fatigue and respiratory symptoms—key features of Long COVID—underscoring the need to assess this period for vocational rehabilitation planning [4,39]. Thus, the six-month mark is a pivotal point where cognitive deficits peak or stabilize, necessitating targeted interventions to mitigate their long-term socioeconomic consequences. Prior studies, however, often focus on acute phases (e.g., Ferrucci et al. [12]) or lack longitudinal depth beyond three months (e.g., Zhou et al. [13]), leaving this critical window underexplored [40].

### 1.5. Objectives and Significance of This Study

This study examines associations between the physical symptom profile (e.g., shortness of breath, severe fatigue, headache), infection history (number of COVID-19 episodes, olfactory/gustatory dysfunction), and self-reported cognitive complaints (memory and attention) in individuals with Long COVID who were assessed six months after their last infection. It additionally explores associations with gender, age, and education. By focusing on a well-defined Long COVID cohort at this critical six-month juncture, this study provides a detailed profile of factors associated with self-reported cognitive complaints, advancing our understanding of Long COVID’s neuropsychological impact and potentially informing tailored clinical interventions.

## 2. Materials and Methods

### 2.1. Study Design

This study employed a cross-sectional design to investigate the relationship between physical symptoms, infection history, demographic factors, and cognitive complaints in individuals with Long COVID. We focused specifically on memory and attention as the core domains of interest because these functions have been most consistently and robustly implicated in previous empirical and neuroimaging studies of Long COVID. Recent systematic reviews and large-scale cohort investigations have identified attention and memory impairments as the most prevalent and functionally disabling cognitive complaints in post-COVID conditions, often serving as sensitive indicators of broader neurocognitive disruption. A 2025 systematic review in Archives of Clinical Neuropsychology synthesized findings from 36 studies and concluded that deficits in attention and memory, alongside executive dysfunction and slowed processing speed, are the most common cognitive sequelae of Long COVID [1]. Structural MRI findings also show that patients with post-COVID cognitive complaints exhibit gray-matter alterations in regions subserving attentional control and episodic memory [2]. Functional connectivity analyses have revealed reduced synchronization within frontoparietal and hippocampal networks associated with short-term memory difficulties [3], while FDG-PET studies demonstrate hypometabolism in areas related to attention and working memory [4]. Moreover, clinical studies using neuropsychiatric batteries have reported the highest prevalence of memory (~27%) and attention (~22%) complaints among Long COVID participants [5]. Collectively, these findings highlight attention and memory as central, measurable, and ecologically valid indicators of post-COVID cognitive dysfunction, justifying their selection as the cognitive domains of focus in the present study. The study population consisted of adults who had a confirmed history of COVID-19 infection (via PCR or clinical diagnosis) and reported persistent symptoms consistent with Long COVID, defined as symptoms lasting beyond four weeks post-infection [4]. Participants were recruited six months after their last confirmed infection to capture the chronic phase of the condition, a timeframe identified as critical for assessing stabilized or persistent cognitive deficits [1]. Participants were selected through convenience sampling from many avenues such as referrals, student groups, and online/social media platforms, notably the Telegram Long COVID Support Group, which boasts a membership of over 10,000 individuals, and psychological clinics in Tehran, Iran, between November 2023 and December 2023. Participants were eligible for inclusion if they (1) were aged 17–71 years, (2) had a confirmed prior COVID-19 infection, (3) reported self-perceived or clinically documented Long COVID symptoms, and (4) were able to provide informed consent after being fully informed about the study.

Exclusion criteria included (1) pre-existing neurological disorders (e.g., dementia, stroke), (2) severe psychiatric conditions (e.g., schizophrenia), and (3) inability to complete cognitive assessments due to language or sensory barriers.

### 2.2. Instrument and Procedure

Data were collected using a multi-component assessment protocol administered online, approximately six months post-infection. The protocol comprised three main instruments:

1. Demographic and Infection History Questionnaire: A structured questionnaire captured participants’ age, gender, education level (categorized as under diploma, undergraduate, BA/BS, MA/MSC, PhD), marital status, number of COVID-19 infections, and presence/absence of olfactory and gustatory dysfunction (including duration in days). This self-reported tool was validated against medical records where available to ensure accuracy.
2. Physical and Neurocognitive Symptom Checklist: A comprehensive checklist, adapted from prior Long COVID studies [2], was used to assess the presence and severity of physical symptoms (e.g., shortness of breath, severe fatigue, headache) and neurocognitive symptoms (e.g., difficulty concentrating, memory lapses) experienced during the past six months. Participants indicated symptom presence on a binary scale (yes/no), with additional items confirming symptom chronicity since infection. To ensure internal consistency within our context, the checklist’s reliability was examined in a pilot test (n = 30) conducted by the research team prior to the main data collection, yielding a Cronbach’s alpha of 0.85, indicating good internal consistency. Details of this reliability analysis are provided in Appendix A (Table A1).
  From the original Long COVID Symptom Inventory, which reports over 200 symptoms across multiple organ systems [2], and given that previous studies (e.g., Graham et al. [20]; Ferrucci et al. [22]) have highlighted a range of persistent neurologic and cognitive complaints, we selected symptoms that were both frequently reported and theoretically related to cognitive functioning. Accordingly, symptoms reflecting fatigue, respiratory difficulty, and pain—known to interfere with attention and memory processes—were prioritized along with core neurocognitive complaints such as concentration, memory, confusion, and decision-making difficulties. This targeted approach ensured interpretability, avoided redundancy, and maintained theoretical and clinical relevance to neurocognitive outcomes in Long COVID.
3. Cognitive Assessment Tools: Cognitive function was evaluated using the Post-COVID Cognitive Impairment Scale, a validated patient-reported outcome measure specifically designed to assess cognitive deficits in individuals with Long COVID, focusing on the key domains of memory and attention [41]. This scale consists of 14 items divided into two subscales: seven items assessing memory (e.g., difficulty remembering tasks, recalling past events, or recognizing familiar individuals) and seven items assessing attention (e.g., difficulty maintaining focus, distinguishing priorities, or concentrating on tasks). Participants rated each item on a five-point Likert scale (1 = Very Little, 5 = Very Much), reflecting the extent of impairment in daily cognitive functioning over the past six months. Total scores range from 14 to 70, with higher scores indicating greater cognitive impairment. The scale’s ecological validity stems from its basis in real-world experiences, and its psychometric properties—established through exploratory and confirmatory factor analyses—demonstrate strong internal consistency (Cronbach’s alpha: memory = 0.81, attention = 0.80) and construct validity, making it suitable for this study’s population [41].

Participants provided informed consent electronically prior to enrollment, and the study was approved by the Ethics Committee of University of Payame Noor Iran, Tehran. Assessments were conducted online via the Porsline platform [42], with individual access links sent to each participant in the study group. Participants completed all questionnaires independently on the platform, and researchers were available online to address any ambiguities or questions regarding the items. Additionally, researchers monitored the completion process in real-time through the platform, providing immediate clarification or additional explanations as needed to ensure accurate responses.

### 2.3. Data Analysis

Data were analyzed using SPSS version 26.0 with a significance level set at *p* < 0.05. Descriptive statistics (mean, standard deviation, frequency, percentage) summarized participant characteristics, symptom prevalence, and cognitive scores. To examine relationships between variables, the following statistical tests were employed:

- Independent *t*-tests: Used to compare mean cognitive scores (memory, attention, total cognitive complaints) between groups defined by binary variables, such as presence/absence of specific physical symptoms (e.g., shortness of breath) or gender. Levene’s test assessed equality of variances, adjusting *t*-test assumptions as needed.
- One-way Analysis of Variance (ANOVA): Applied to assess differences in cognitive scores across education levels, followed by Scheffe post hoc tests to identify specific group differences when ANOVA yielded significant results.
- Pearson Correlation: Conducted to explore associations between continuous variables, such as age, number of COVID-19 infections, duration of olfactory/gustatory dysfunction, and cognitive scores.
- Chi-squared Tests: Associations between categorical variables were examined using Pearson’s χ^2^. Gender (binary) and education (five levels: under diploma, undergraduate, BA/BS, MA, PhD; not dichotomized) were each tested against the presence (yes/no) of neurocognitive complaints. Degrees of freedom for education tests were df = 4. Two-tailed α = 0.05.
- Multiple Linear Regression: Conducted to evaluate the combined predictive contribution of demographic variables (gender, age, education) and clinical factors (number of physical symptoms, duration of olfactory/gustatory dysfunction, and number of COVID-19 infections) to total cognitive complaints. Regression assumptions (normality, linearity, multicollinearity, and homoscedasticity) were checked and met. Statistical significance for all models was set at *p* < 0.05.

## 3. Results

### 3.1. Sample Characteristics

This section provides an overview of the 212 participants assessed six months post-COVID-19 infection, including age, gender, education, marital status, quarantine history, and infection history. Table 1 summarizes these characteristics. The study sample comprised 212 individuals with Long COVID (N = 212). The mean age was 39.7 years (SD = 10.5), and 67.9% of participants were female. Education levels were distributed as follows: Bachelor’s degree 28.3%, Master’s degree 35.4%, PhD 8.0%, and the remainder with lower formal education. Approximately 42.5% of participants reported olfactory/gustatory dysfunction during the acute phase. Participants were recruited and assessed approximately six months after their last confirmed infection. Full participant characteristics and symptom frequencies are presented in Table 1.

The sample had a mean age of 39.7 years (SD = 10.5), ranging from 17 to 71 years, with a predominantly female composition (67.9%). Education levels were diverse, with the majority holding a master’s degree (35.4%) or bachelor’s degree (28.3%). Most participants were married (62.3%), while 36.5% were single (including never married and divorced individuals). Retrospectively, about half of the sample (50.7%) reported quarantine during the acute phase of their infection, and more than half (51.7%) experienced olfactory and gustatory dysfunction during that period (Table 1).

### 3.2. Prevalence of Symptoms at Six Months

The frequency of physical and neurocognitive symptoms reported six months post-infection is detailed below. Physical symptoms included shortness of breath, severe infection, high fever, abdominal pain, headache, dizziness, chronic pain, chronic fatigue, sleep disturbance, and severe fatigue after mild physical activity. It should be noted that severe infection and high fever are symptoms typically experienced during the acute phase of COVID-19; therefore, these two items were assessed retrospectively, asking participants to report whether they had experienced them during the active stage of infection. Neurocognitive symptoms encompassed difficulty concentrating, slowed thinking, confusion, memory lapse, feeling disoriented, difficulty making decisions, and difficulty retaining new information. Table 2 consolidates their prevalence.

Among physical symptoms, severe fatigue after mild activity (23.2%) and chronic fatigue (22.7%) were the most prevalent, followed by headache (19.0%). Other physical symptoms ranged from 5.7% (severe infection) to 13.3% (high fever). Neurocognitive symptoms were notably common, with difficulty retaining new information (57.9%) and difficulty concentrating (52.1%) affecting over half the sample, and memory lapse reported by 47.9%. Neurocognitive symptoms ranged from 19.4% (feeling disoriented) to 57.9% (difficulty retaining new information). These findings highlight a substantial burden of cognitive-related symptoms at six months, with fatigue and cognitive difficulties predominating (Table 2).

### 3.3. Associations with Cognitive Complaints

This section examines the relationships between physical symptoms, Neurocognitive symptoms, and demographic factors (age, gender, education) six months post-COVID-19 infection. Key findings are presented in three tables below, with detailed statistical outputs available in the Appendix A.

#### 3.3.1. Physical Symptoms and Cognitive Subdomains

Table 3 examines the associations between physical symptoms and the memory impairment and attention impairment subdomains of cognitive complaints, as these core components of cognition are consistently among the most affected in individuals with Long COVID. The Total Cognitive Complaints score was calculated as the sum of the two primary subdomain scores—memory impairment and attention impairment—representing the overall severity of self-reported cognitive difficulties.

Shortness of breath was associated with higher total cognitive complaints (M_yes = 38.44 vs. M_no = 33.92; t(209) = 2.19, *p* = 0.030) and higher attention impairment scores (M_yes = 20.67 vs. M_no = 17.62; t(209) = 2.33, *p* = 0.021), but not with memory impairment (*p* = 0.171). Headache was associated with higher total complaints (M_yes = 36.77 vs. M_no = 33.73; t(209) = 2.07, *p* = 0.040) and greater memory impairment (M_yes = 17.82 vs. M_no = 16.10; t(209) = 2.27, *p* = 0.024), but not attention (*p* = 0.161). Severe fatigue after mild activity was associated with higher total complaints, greater memory impairment and greater attention impairment (all *p* < 0.05). Dizziness showed trends toward higher total complaints (*p* = 0.078) and memory impairment (*p* = 0.062). No significant associations were observed for abdominal pain, chronic pain, chronic fatigue, or sleep disturbance (all *p* > 0.05).

Effect sizes for significant comparisons were in the small-to-moderate range. For example, shortness of breath was associated with a moderate effect on total cognitive complaints (Cohen’s d = 0.54; Hedges’ g = 0.54). Headache showed a small-to-moderate effect on total complaints (d = 0.36), and severe fatigue after mild activity showed a small-to-moderate effect on total complaints (d = 0.46).

#### 3.3.2. Neurocognitive Symptoms and Demographic Factors

Seven neurocognitive complaints—difficulty concentrating, slowed thinking, confusion, memory lapse, feeling disoriented, difficulty making decisions, and difficulty retaining new information—were assessed for prevalence and associations with demographic variables six months post-infection. Table 4 summarizes the prevalence of these complaints across the sample (n = 212) and reports bivariate test results for age (independent-samples *t*-tests) and gender/education (chi-square tests). This overview highlights key demographic patterns—for example, several cognitive complaints were more common among younger participants, some differed by gender, and others varied across education levels.

Several neurocognitive complaints showed significant demographic associations. Specifically, confusion (*p* = 0.001) and difficulty making decisions (*p* = 0.021) were associated with younger age (see Table 6). Difficulty making decisions also differed by gender (*p* = 0.008); inspection of within-gender proportions indicates a higher within-gender prevalence among males (see Table 5). Difficulty retaining new information was more prevalent among females (*p* = 0.034; see Table 5). Difficulty concentrating varied across education levels (χ^2^(4) = 22.38, *p* = 0.001) and displayed a non-linear distribution across education categories (see Table 7).

**Table 5:** Gender Differences in Neurocognitive Symptoms at Six Months Post-Infection (n = 212).

Neurocognitive Symptom	Prevalence, n (%)	Female, n (%)	Male, n (%)	Chi-Square	*p*-Value
Difficulty retaining new information	122 (57.8%)	90 (62.5%)	32 (47.1%)	4.51	0.034 *
Difficulty concentrating	110 (52.1%)	73 (50.7%)	37 (54.4%)	0.66	0.613
Memory lapse	101 (47.9%)	69 (47.9%)	32 (47.1%)	0.01	0.907
Slowed thinking	73 (34.6%)	52 (36.1%)	21 (30.9%)	0.26	0.455
Difficulty making decisions	59 (28.05)	32 (22.2%)	27 (39.7%)	7.03	0.008 *
Confusion	44 (20.9%)	26 (18.1%)	18 (26.5%)	1.99	0.158
Feeling disoriented	41 (19.4%)	28 (19.4%)	13 (19.1%)	0.01	0.955

Notes: Prevalence (second column) is reported as n (% of the total sample; N = 212). The Male and Female columns report counts and within-gender percentages (n; % of males or % of females); Male N = 68 (32.1%), Female N = 144 (67.9%). Chi-square tests (χ^2^(1)) assess associations between gender and symptom presence. * *p* < 0.05.

**Table 6:** Age Differences in Neurocognitive Symptoms at Six Months Post-Infection (n = 212).

Neurocognitive Symptom	Prevalence, n (%)	Mean Age (SD), No	Mean Age (SD), Yes	t-Value	df	*p*-Value	Mean Difference	Cohen’s d
Difficulty retaining new information	122 (57.8)	38.5 (9.6)	40.6 (11.1)	−1.44	210	0.151	−2.09	—
Difficulty concentrating	110 (52.1)	41.1 (10.3)	38.4 (10.5)	1.90	210	0.058	2.73	0.26
Memory lapse	101 (47.9)	40.5 (11.2)	38.9 (9.6)	1.11	209.08	0.268	1.59	—
Slowed thinking	73 (34.6)	39.6 (10.5)	40 (10.4)	−0.27	210	0.790	−0.40	—
Difficulty making decisions	59 (28.0)	40.7 (11)	37.0 (8.6)	2.32	210	0.021 *	3.68	0.36
Confusion	44 (20.9)	40.8 (10.9)	35.6 (7.4)	3.66	97.68	0.001 *	5.12	0.50
Feeling disoriented	41 (19.4)	40.2 (10.4)	37.7 (10.5)	1.34	210	0.183	2.43	—

Notes: Prevalence is reported as n (% of N = 212). Mean age (SD) is shown for participants without (No) and with (Yes) each symptom. *t*-tests compare mean ages; Welch’s *t*-test with adjusted degrees of freedom (df) was used where Levene’s test indicated unequal variances (Levene *p* < 0.05 for confusion and memory lapse). Mean difference is reported as M_no − M_yes (years). * *p* < 0.05 indicates statistical significance.

**Table 7:** Education Level and Neurocognitive Symptoms Prevalence at Six Months Post-Infection (n = 212).

Neurocognitive Symptom		Under Diploma (n = 25)	Under Graduate (n = 35)	BA/BS (n = 60)	MA (n = 75)	PhD (n = 17)	Chi-Square	*p*-Value
Difficulty Retaining New Information	No	11 (44.0%)	12 (34.3%)	26 (43.3%)	36 (48.0%)	5 (29.4%)	3.1	0.5
	Yes	14 (56.0%)	23 (65.7%)	34 (56.7%)	39 (52.0%)	12 (70.6%)		
Difficulty Concentrating	No	10 (40.0%)	9 (25.7%)	43 (71.7%)	34 (45.3%)	6 (35.3%)	22.4	0.0 *
	Yes	15 (60.0%)	26 (74.3%)	17 (28.3%)	41 (54.7%)	11 (64.7%)		
Memory lapse	No	10 (40.0%)	15 (42.9%)	37 (61.7%)	39 (52.0%)	10 (4.7%)	5.2	0.3
	Yes	15 (60.0%)	20 (57.1%)	23 (38.3%)	36 (48.0%)	7 (3.3%)		
Slowed Thinking	No	14 (56.0%)	24 (68.6%)	41 (68.3%)	51 (68.0%)	9 (52.9%)	2.8	0.6
	Yes	11 (44.0%)	11 (31.4%)	19 (31.7%)	24 (32.0%)	8 (47.1%)		
Difficulty Making Decisions	No	19 (76.0%)	21 (60.0%)	43 (71.7%)	58 (77.3%)	12 (70.6%)	3.8	0.4
	Yes	6 (24.0%)	14 (40.0%)	17 (28.3%)	17 (22.7)	5 (29.4%)		
Confusion	No	17 (68.0%)	27 (77.1%)	48 (80.0%)	63 (84.0%)	13 (76.5%)	3.2	0.5
	Yes	8 (32.0%)	8 (22.9%)	12 (20.0%)	12 (16.0%)	4 (23.5%)		
Feeling Disoriented	No	17 (68.0%)	27 (77.1%)	51 (85.0%)	64 (85.3%)	12 (70.6%)	5.7	0.2
	Yes	8 (32.0%)	8 (22.9%)	9 (15.0%)	11 (14.7%)	5 (29.4%)		

Notes: Cells report raw counts and within-group percentages (n; % within each education category). Education levels: Under Diploma (<high school), Undergraduate (some college/no degree), BA/BS (Bachelor), MA (Master), and PhD (Doctorate). Data are drawn from the Physical and Neurocognitive Symptom Checklist, in which responses were dichotomous (Yes/No) for symptoms reported at six months post-infection. Associations between education level and symptom presence/absence were tested using chi-square tests (χ^2^; df = 4), appropriate for categorical outcomes; expected cell counts met statistical assumptions. *p*-values are two-tailed and unadjusted for multiplicity (* *p* < 0.05).

To provide a more detailed examination of these demographic associations, the following tables present stratified analyses by gender, age, and education. Table 5 evaluates gender differences in the prevalence of each neurocognitive complaint; counts and within-gender percentages are reported for males and females, and associations are tested using chi-square tests (χ^2^; df = 1). Table 6 examines age-related patterns by comparing mean ages (M, SD) of participants with versus without each symptom; independent-samples *t*-tests are reported (mean difference = M_no − M_yes), with Welch’s correction and adjusted degrees of freedom applied where Levene’s test indicated unequal variances. Table 7 investigates the distribution of symptoms across five education categories (Under Diploma, Undergraduate, BA/BS, MA/MSC, PhD); cells show counts and within-education percentages, and associations are tested with chi-square analyses (χ^2^; df = 4). Together these tables provide a granular perspective on how gender, age, and education relate to neurocognitive complaints in this cohort.

Among the seven neurocognitive symptoms, two showed significant gender differences. Difficulty making decisions differed by gender (χ^2^(1) = 7.03, *p* = 0.008); when examined as within-gender proportions, this symptom was more prevalent among males (27/68 = 39.7% of males vs. 32/144 = 22.2% of females). Difficulty retaining new information was more prevalent among females both in absolute and within-gender terms (χ^2^(1) = 4.51, *p* = 0.034; 90/144 = 62.5% of females vs. 32/68 = 47.1% of males). No significant gender differences were observed for difficulty concentrating (χ^2^(1) = 0.66, *p* = 0.613), slowed thinking (χ^2^(1) = 0.26, *p* = 0.455), confusion (χ^2^(1) = 1.99, *p* = 0.158), memory loss (χ^2^(1) = 0.01, *p* = 0.907), or feeling disoriented (χ^2^ (1) = 0.01, *p* = 0.955).

Two neurocognitive symptoms showed significant age differences. Participants reporting confusion were younger (M_yes = 35.6, SD = 7.4 vs. M_no = 40.8, SD = 10.92; t(97.68) = 3.66, *p* = 0.001; mean difference = 5.12 years; Cohen’s d = 0.50). Similarly, participants reporting difficulty making decisions were younger (M_yes = 37.0, SD = 8.6 vs. M_no = 40.7, SD = 11; t(210) = 2.32, *p* = 0.021; mean difference = 3.68 years; Cohen’s d = 0.36). A trend was observed for difficulty concentrating, with younger participants reporting higher scores (M_yes = 38.4 vs. M_no = 41.1; t(210) = 1.90, *p* = 0.058; Cohen’s d = 0.26). No significant age differences were found for slowed thinking, memory lapse, feeling disoriented, or difficulty retaining new information (all *p* > 0.05). Note: Welch’s *t*-test was used where Levene’s test indicated unequal variances (df reported accordingly).

Here, we report neurocognitive outcomes at two complementary levels, aligned with the measurement properties of the instruments to avoid ambiguity. Table 7 draws on the Physical and Neurocognitive Symptom Checklist, in which responses are dichotomous (Yes/No). It summarizes the prevalence of each complaint across education levels (frequencies and percentages) and tests associations between education and the presence/absence of symptoms using chi-square (χ^2^) analyses—appropriate for categorical outcomes. In contrast, Table 8 uses the Post-COVID Cognitive Impairment Scale, a validated patient-reported outcome specifically designed to assess cognitive deficits in individuals with Long COVID, focusing on the key domains of memory and attention. Here, we analyze continuous severity scores (total cognitive complaints, memory, attention) across the five education levels with one-way ANOVA; homogeneity of variances is evaluated with Levene’s test, and when the omnibus F is significant, Scheffé post hoc comparisons identify the specific group differences. Taken together, Table 7 addresses the question “Are these complaints more common in some education groups?”, while Table 8 addresses “Are mean severity levels higher in some groups?”, providing complementary—not redundant—evidence.

Education level was significantly associated with difficulty concentrating (χ^2^(4) = 22.4, *p* < 0.001). As shown in Table 7, the within-group prevalence of difficulty concentrating varied across education levels: Undergraduate = 26/35 (74.3%), PhD = 11/17 (64.7%), Under Diploma = 15/25 (60.0%), MA = 41/75 (54.7%), and BA/BS = 17/60 (28.3%).

Thus, BA/BS holders showed the lowest within-group prevalence, whereas undergraduates showed the highest.

No significant associations were found between education level and the other six neurocognitive symptoms: slowed thinking (χ^2^(4) = 2.8, *p* = 0.6), confusion (χ^2^(4) = 3.2, *p* = 0.5), memory lapse (χ^2^(4) = 5.2, *p* = 0.3), feeling disoriented (χ^2^(4) = 5.7, *p* = 0.2), difficulty making decisions (χ^2^(4) = 3.8, *p* = 0.4), or difficulty retaining new information (χ^2^(4) = 3.1, *p* = 0.5). These findings indicate that education level was primarily associated with difficulty concentrating in this cohort, with minimal associations for other neurocognitive complaints.

Across five education levels, omnibus ANOVAs indicated statistically significant group differences for total cognitive complaints, memory, and attention (Table 8). Effects were small-to-moderate (η^2^ ≈ 0.06–0.08). Scheffé post hoc tests showed that participants with Under Diploma education reported higher total and memory impairment than those with a BA/BS (ΔMs = 6.16 and 3.30, respectively). For attention, the Undergraduate group (some college/no degree) scored higher than both BA/BS and MA/MSC groups (ΔMs = 3.87 and 3.78). No other pairwise contrasts reached statistical significance after Scheffé adjustment. These findings complement the chi-square analyses of symptom prevalence (Table 7) by demonstrating education-related differences in severity of cognitive complaints.

Combined summary (Table 7 and Table 8). Education level showed a selective association with neurocognitive outcomes at six months. In the prevalence analyses (Table 7; Yes/No checklist, χ^2^ tests), only difficulty concentrating varied by education—χ^2^(4) = 22.38, *p* = 0.001—with within-group prevalence highest in Undergraduate (26/35, 74.3%) and PhD (11/17, 64.7%), intermediate in Under Diploma (15/25, 60.0%) and MA/MSC (41/75, 54.7%), and lowest in BA (17/60, 28.3%). No other symptom (slowed thinking, confusion, memory lapse, feeling disoriented, difficulty making decisions, difficulty retaining new information) was significantly associated with education (all ps ≥ 0.221). In the severity analyses (Table 8; P-CCIS continuous scores, one-way ANOVA with Levene and Scheffé), omnibus tests were significant for total cognitive complaints, memory, and attention—F(4,207) = 4.70, *p* = 0.001, η^2^ = 0.083; F(4,207) = 3.25, *p* = 0.013, η^2^ = 0.059; and F(4,207) = 4.64, *p* = 0.001, η^2^ = 0.083, respectively. Post hoc comparisons indicated Under Diploma > BA/BS for total complaints (Δ*M* = 6.16, *p* = 0.043) and memory (Δ*M* = 3.30, *p* = 0.035), and Undergraduate > BA/MA for attention (Δ*M*s = 3.87/0.016; 3.78/0.014); no other pairwise differences reached significance. Taken together, education relates chiefly to the presence of difficulty concentrating and to higher severity of cognitive complaints—particularly among lower (Under Diploma) and some-college/no-degree (Undergraduate) groups—while BA/MA tend to show lower burden; effects are small-to-moderate in magnitude (η^2^ ≈ 0.06–0.08).

#### 3.3.3. Infection History and Cognitive Outcomes

Participants retrospectively reported infection-related experiences during the acute phase of COVID-19, including severe infection, high fever, olfactory and gustatory dysfunction, duration of olfactory and gustatory dysfunction, and frequency of COVID-19 infections. Data on seizures and days of home quarantine were also collected but are not analyzed here due to limited statistical power or incomplete data. Table 9 summarizes the prevalence of these infection characteristics and their associations with cognitive complaints and its subdomains (memory and attention) six months after the last infection, assessed using *t*-tests for binary variables (severe infection, high fever, olfactory/gustatory dysfunction) and Pearson correlations for continuous variables (duration of dysfunction, number of infections).

Severe infection showed a trend toward higher total cognitive complaints (No: *M* = 34.1, SD = 8.6; Yes: *M* = 37.1, SD = 4.46; t(16.45) = −2.06, *p* = 0.055; Cohen’s d ≈ 0.35), but the severe-infection group was small (Yes n = 12) and the difference did not reach conventional significance. Olfactory/gustatory dysfunction was associated with higher memory-impairment scores (No: *M* = 15.8, SD = 4.2; Yes: *M* = 17.0, SD = 4.43; t(209) = −2.11, *p* = 0.036; d ≈ 0.29). Attention impairment scores did not differ by severe infection, high fever, or olfactory/gustatory dysfunction (all *p* ≥ 0.236). Pearson correlations (two-tailed) were used to examine continuous infection characteristics. No significant correlations were observed between continuous infection variables and cognitive outcomes. Specifically, duration of olfactory/gustatory dysfunction (n = 210) showed a very small, non-significant positive association with total cognitive complaints (*r* = 0.029, *p* = 0.675) and with memory impairment scores (r = 0.092, *p* = 0.185), and a very small, non-significant negative association with attention impairment scores (*r* = −0.028, *p* = 0.682). Number of COVID-19 infections (n = 211) showed weak, non-significant positive associations with total complaints (*r* = 0.068, *p* = 0.324) and memory impairment (r = 0.112, *p* = 0.106) and was essentially uncorrelated with attention impairment (r = 0.017, *p* = 0.811). All observed correlations were small in magnitude (|*r*| < 0.12) and did not reach statistical significance, indicating no meaningful linear relationships in this sample. We tested only linear associations (Pearson); non-linear associations were not examined.

#### 3.3.4. Multivariable Regression Analysis

To identify the combined contribution of demographic and clinical factors to total cognitive complaints, a multiple linear regression analysis was conducted including gender, age, education level, number of physical symptoms, duration of olfactory/gustatory dysfunction, and number of infections as predictors. (Table 10).

Regression assumptions (normality, multicollinearity, and homoscedasticity) were checked and met. Statistical significance for all models was set at *p* < 0.05.

As shown in Table 10, the overall regression model explained approximately 6.7% of the variance in total cognitive complaints (R^2^ = 0.07, Adjusted R^2^ = 0.04).

Among the predictors, both education level (*β* = –0.18, *p* = 0.0) and number of physical symptoms (*β* = 0.19, *p* = 0.0) were significant.

Specifically, higher educational attainment was associated with fewer cognitive complaints, while a greater number of physical symptoms predicted higher complaint scores.

Gender, age, duration of olfactory/gustatory dysfunction, and number of infections were not statistically significant predictors.

## 4. Discussion

### 4.1. Main Findings and Contextualization

This study examined the prevalence and correlates of self-reported cognitive complaints in 212 adults assessed approximately six months after their most recent SARS-CoV-2 infection. Using the Post-COVID Cognitive Impairment Scale [41], we observed that memory- and attention-related complaints were prominent features of Long COVID in this cohort, with nearly half reporting memory problems (47.9%) and a majority reporting difficulty retaining new information (57.8%) and concentrating (52.1%). In parallel, a substantial burden of ongoing physical and fatigue-related symptoms was present: severe fatigue after mild activity (23.2%) and chronic fatigue (22.7%) were among the more commonly reported somatic complaints.

These prevalence estimates align with prior clinic- and population-based work documenting persistent fatigue and cognitive symptoms in Long COVID [2,4,26,40]. For example, multi-center and international surveys [19,43,44], have reported substantial rates of post-infectious fatigue and “brain fog” at 6–12 months, though absolute prevalence varies across samples and recruitment strategies [2,40,45]. The prominent reporting of severe post-exertional fatigue in our sample is consistent with descriptions of post-exertional malaise as a feature of Long COVID and related post-viral syndromes [25,46].

Importantly, we found that certain physical symptoms tended to co-occur with higher self-reported cognitive complaints: participants endorsing shortness of breath, headache, or severe fatigue after mild activity reported greater cognitive difficulties than those not reporting these symptoms. These are associative findings and should not be interpreted as evidence of causation. They do, however, complement emerging neurobiological evidence—such as altered brain connectivity and markers of neuroinflammation—that may help explain why some patients experience persistent cognitive symptoms following SARS-CoV-2 infection [32,47,48,49,50]. Finally, because our cognitive assessment is based on self-report, the present results describe perceived cognitive difficulties (complaints) rather than objectively measured cognitive impairment; objective neuropsychological testing and neuroimaging are required to determine the extent and mechanisms of any objectively measurable deficits in Long COVID.

For detailed prevalence estimates and full statistical outputs, see Results.

### 4.2. Physical Symptoms and Cognitive Complaints

Shortness of breath was associated primarily with higher self-reported attention complaints in our sample, while its relation to memory complaints was weaker; prior work has similarly linked dyspnea and reduced cardiorespiratory function to cognitive processing difficulties [51,52]. This discrepancy with some reports that describe broader post-COVID cognitive effects [31] may reflect differences in sample composition and illness severity—only 5.7% of our participants reported severe acute infection—whereas other cohorts included larger proportions of hospitalized patients [53,54].

Headache in our cohort was specifically associated with greater self-reported memory complaints but not with attention complaints, a pattern that echoes prior reports of neurological sequelae in Long COVID [37,55,56]. Nordvig et al. [57] identified headache as a frequent precursor to cognitive complaints, potentially reflecting neuroinflammatory processes [57]. Our observed headache prevalence is consistent with ranges reported in systematic reviews (≈20–30%), although the apparent domain-specific cognitive impact of headache (memory vs. attention) suggests that underlying mechanisms deserve targeted investigation [27,38,58].

Notably, severe fatigue after mild activity showed the most consistent associations with elevated complaints in both memory and attention domains, consistent with Appelman et al. (2024), who linked muscle abnormalities and post-exertional malaise to cognitive deficits [25]. This finding underscores the overlap between Long COVID and chronic fatigue syndrome [59], suggesting shared pathophysiological pathways, such as mitochondrial dysfunction or immune dysregulation [35,60]. In contrast, chronic fatigue alone showed no significant cognitive association, possibly indicating distinct fatigue subtypes within Long COVID [61]. Objective cognitive testing and physiological measures are needed to disentangle mechanisms and confirm whether these self-reported complaints correspond to measurable deficits.

### 4.3. Neurocognitive Symptoms and Demographic Correlates

The prominence of self-reported neurocognitive complaints in our cohort—including difficulty retaining new information, difficulty concentrating, and memory lapses—reinforces descriptions of a neuro-Long COVID phenotype reported elsewhere [2,20]. Differences in prevalence and symptom detail across studies likely reflect variation in assessment methods and sampling strategies; for example, Bahmer et al. [40] report high rates of neurological complaints in a multicenter sample, while our use of a targeted cognitive scale may capture different patterns of complaint [41]. Large surveys have likewise emphasized the pervasiveness of “brain fog” in diverse cohorts [39].

Demographic analyses yielded nuanced patterns rather than uniform effects. We did not observe an overall gender difference in total cognitive complaints, a finding that contrasts with reports of greater neurological symptom burden among females in some studies [7]. However, more detailed inspection showed higher reporting among females for particular neurocognitive items—most notably difficulty making decisions and difficulty retaining new information (see Table 5)—suggesting that sex-related differences may be symptom-specific rather than global. These patterns invite consideration of biopsychosocial explanations (e.g., hormonal factors, differential health-seeking or symptom-reporting behavior), which warrant targeted investigation [62].

Age effects were complex. While overall cognitive complaint burden did not mirror patterns reported in some cohorts where age and acute-phase severity predicted worse outcomes [4], we observed that certain complaints (e.g., confusion; difficulty making decisions) were reported more frequently by younger participants, a pattern also noted in studies of non-hospitalized patients [63]. Such findings may reflect cohort differences in exposure, stressors, or immune response rather than a straightforward age-driven vulnerability [10,21,64,65,66,67].

Consistent with the cognitive-reserve account [15], one-way ANOVAs on continuous Post-COVID Cognitive Impairment Scale [41], showed small-to-moderate education differences (η^2^ ≈ 0.06–0.08): participants with Under Diploma education scored higher than BA/BS on total complaints and memory impairment, and the Undergraduate group (some college/no degree) scored higher than BA/MA on attention impairment. By contrast, χ^2^ tests on the dichotomous checklist indicated that education was associated only with the prevalence of difficulty concentrating—highest in Undergraduate and PhD and lowest in BA/BS—while the other six complaints did not vary by education. Taken together, education appears to shape both the likelihood of reporting certain complaints and the severity of cognitive difficulties, with BA/MA generally showing the lowest burden; this pattern aligns with the view that cognitive reserve buffers post-infectious cognitive sequelae [15] and also suggests a role for social determinants in symptom expression and reporting [68]. Overall, these demographic associations emphasize heterogeneity in Long COVID’s neurocognitive presentation: sex, age, and education do not uniformly predict cognitive complaints but may influence the likelihood of specific symptom types. As with other aspects of our study, these observations are associative and based on self-report; replication with objective cognitive testing and larger, more diverse samples are required to clarify mechanisms and clinical implications.

Extending these bivariate findings, the multivariable regression analysis provided a more integrated perspective on how demographic and clinical variables jointly contributed to the overall burden of cognitive complaints. The model accounted for approximately 7% of the variance in total complaint scores (Adjusted R^2^ = 0.04), revealing that education level and number of physical symptoms were the only significant predictors. Specifically, higher educational attainment predicted fewer cognitive complaints, while a greater number of physical symptoms predicted higher complaint scores. These findings are compatible with a cognitive-reserve interpretation—where higher educational attainment buffers the cognitive impact of post-viral conditions [15]—and are congruent with neuroimaging evidence of post-COVID brain changes that may underlie persistent cognitive difficulties [16]. Conversely, the positive association between somatic symptom load and cognitive complaints supports emerging evidence linking systemic symptom severity—particularly fatigue and dyspnea—to self-perceived cognitive dysfunction [25,46,51]. The lack of significant effects for age, gender, infection frequency, or sensory dysfunction duration further indicates that the persistence of cognitive difficulties may be better explained by the cumulative physical symptom burden and individual protective factors (e.g., cognitive reserve) rather than by acute-phase or demographic characteristics alone.

Recent neuroimaging and neurophysiological studies provide converging support for these self-reported cognitive patterns, revealing measurable alterations in brain structure and function among individuals with Long COVID. Functional MRI and EEG studies have documented disrupted frontoparietal connectivity, reduced cortical excitability, and abnormal oscillatory activity associated with attentional and working-memory deficits [16,48,49,50,69,70]. Similarly, TMS-based studies have reported reduced cortical excitability and altered plasticity in patients with Long COVID, suggesting possible neurophysiological correlates of post-viral fatigue and cognitive dysfunction [71,72].

Although our study relied on self-reported measures, the correspondence between perceived cognitive difficulties and objective neurofunctional abnormalities underscores the validity of patient-reported cognitive complaints as indicators of underlying neurobiological change. Integrating self-report tools with neuroimaging and electrophysiological methods in future research will help delineate the neural correlates of these persistent cognitive symptoms and clarify their clinical relevance.

### 4.4. Clinical and Research Implications

The substantial burden of self-reported cognitive complaints observed here—particularly in relation to fatigue and headache—underscores the clinical need for systematic screening and symptom-directed care. Clinicians in outpatient and primary-care settings could integrate brief, validated screening instruments (including self-report tools such as the Post-COVID Cognitive Impairment Scale [41]) to identify patients who may benefit from further assessment or referral [63]. For individuals with prominent cognitive complaints, stepped care pathways that include fatigue-management programs [73], pacing and graded activity approaches, and tailored cognitive rehabilitation [36,74] should be considered within multidisciplinary services. Referral pathways for neuropsychiatric, sleep, and cardiopulmonary evaluation may also be warranted when symptoms cluster with somatic signs.

The lack of association between infection frequency or sensory-dysfunction duration and cognitive complaints in our sample suggests that symptom persistence may not be simply explained by acute severity, a perspective compatible with earlier syntheses of post-COVID sequelae [3]. Our findings also challenge assumptions of uniform Long COVID profiles across populations and underscore the need for culturally sensitive research approaches [18,75]. The absence of a clear cognitive impact of severe acute infection in our cohort contrasts with some reports from hospitalized samples [45], highlighting the value of focused investigation in non-hospitalized cohorts [21]. Mechanistic neuroimaging studies (e.g., fMRI) could help elucidate pathways linking fatigue and cognitive complaints [16,17], while longitudinal designs are needed to clarify symptom trajectories and persistence over time [4].

From a research perspective, our findings signal several priorities. First, intervention trials are needed to test whether targeted rehabilitative and symptom-management approaches reduce self-reported cognitive complaints and improve function. Second, mechanistic studies that combine objective neuropsychological testing, neuroimaging (e.g., fMRI), and physiological/biomarker assessments are required to delineate the biological substrates linking systemic symptoms (e.g., post-exertional fatigue, dyspnea) with cognitive complaints. Third, longitudinal cohorts with standardized cognitive measures would help clarify symptom trajectories and distinguish transient from persistent deficits. Finally, research should attend to sampling diversity and cultural context—heterogeneity in recruitment and assessment likely contributes to inconsistent prevalence estimates across studies—and prioritize non-hospitalized and community samples that reflect the broader population affected by Long COVID.

### 4.5. Limitations and Future Directions

Our study’s focus on an Iranian population introduces limitations in generalizability, as cultural, socioeconomic, and healthcare system factors—such as access to medical resources, health literacy, or dietary habits—may differ substantially from Western cohorts or other global populations. While this specificity strengthens the study’s relevance to Middle Eastern contexts, it underscores the need for cross-cultural validation to determine whether our observed cognitive burden patterns hold universally. Additionally, the exclusion of participants with pre-existing cognitive impairments, though methodologically justified to enhance internal validity and isolate Long COVID effects, may have led to an underestimation of the true population-level burden. This decision potentially overlooks how pre-existing conditions could interact with or exacerbate post-COVID cognitive symptoms, a critical consideration for real-world clinical applicability.

Although the overall sample size (N = 212) was adequate for the study design and analyses, the use of a convenience sampling approach and the relatively small size of certain symptom-specific subgroups—such as participants reporting sleep disturbance (n = 16)—may have limited the representativeness of the sample and reduced statistical power to detect subtle associations. This challenge is not unique to our work but reflects a broader issue in Long COVID research, where symptom heterogeneity and variable prevalence complicate robust analyses.

Moreover, not all dimensions of physical and neurocognitive symptoms were assessed, as the study evaluated ten physical and seven neurocognitive symptoms, with the cognitive component primarily focused on memory- and attention-related complaints. Future investigations should include broader domains, such as processing speed, executive function, language, and visuospatial abilities, to achieve a more comprehensive understanding of post-COVID cognitive outcomes.

Similarly, reliance on self-reported data introduces potential recall bias, particularly for symptoms like memory lapses or difficulty concentrating, which participants may over- or under-report based on subjective perception or psychological state at the time of assessment. The cross-sectional design further restricts our ability to infer causality or track symptom evolution over time, leaving questions about the chronicity and trajectory of cognitive impairments unanswered.

Future research should prioritize several avenues to address these gaps. First, validating our findings across diverse populations—spanning different ethnicities, healthcare infrastructures, and socioeconomic backgrounds—would enhance generalizability and clarify the role of contextual factors in Long COVID outcomes. Second, incorporating objective cognitive measures, such as standardized neuropsychological tests (e.g., Montreal Cognitive Assessment) or neuroimaging (e.g., fMRI), could provide a more precise assessment of impairment and reduce reliance on subjective reporting. Third, longitudinal studies tracking symptom progression over months or years would elucidate whether these cognitive deficits are transient, persistent, or progressive, informing both prognosis and intervention timing. Finally, larger and more representative samples, along with broader assessment of physical and cognitive domains, would yield a more complete picture of Long COVID’s cognitive toll and support the development of tailored therapeutic strategies.

## 5. Conclusions

This study demonstrates that self-reported cognitive complaints—particularly difficulties with memory, concentration, and retaining new information—remain prevalent six months after SARS-CoV-2 infection, even among non-hospitalized individuals. These findings highlight the lasting cognitive and functional burden of Long COVID and its close association with physical symptoms such as post-exertional fatigue, headache, and dyspnea.

The multivariable regression model explained about 7% of the variance in total cognitive complaints, identifying education level and number of physical symptoms as the only significant predictors. Higher educational attainment was associated with fewer cognitive complaints, whereas a greater burden of physical symptoms predicted higher complaint scores. Other demographic or clinical variables—including gender, age, infection frequency, and duration of sensory dysfunction—were not significant predictors.

Overall, these findings support a cognitive-reserve interpretation and emphasize the importance of symptom burden as a key determinant of perceived cognitive difficulties. Routine cognitive screening and individualized rehabilitation—integrating fatigue management, pacing strategies, and targeted cognitive interventions—should be incorporated into post-COVID care. Future longitudinal and multimodal studies combining self-report, neuropsychological, and neuroimaging measures are essential to clarify mechanisms and guide effective, patient-centered interventions.

## Figures and Tables

**Table 1:** Demographic and Infection History Characteristics of the Study Sample (n = 212).

Characteristic	Value
Age (years)	
Mean (SD)	39.7 (10.5)
Median	39.0
Range	17–71
Gender, n (%)	
Female	144 (67.9%)
Male	68 (32.1%)
Education, n (%)	
Under Diploma	25 (11.8%)
Undergraduate (Diploma)	35 (16.5%)
Bachelor’s Degree (BA/BS)	60 (28.3%)
Master’s Degree (MA/MSC)	75 (35.4%)
Doctoral Degree (PhD)	17 (8.0%)
Marital Status, n (%)	
Single	77 (36.5%)
Married	132 (62.3%)
Widowed	3 (1.4%)
Quarantine History, n (%)	
Quarantined	107 (50.7%)
Number of COVID-19 Infections, n (%)	
1	119 (56.1%)
2 or more	93 (43.9%)
Days of Quarantine (for those quarantined)	
Mean (SD)	15.18 (7.9)
Olfactory/Gustatory Dysfunction, n (%)	
Present	109 (51.7%)

Notes: Data are self-reported. Some analyses use reduced n due to missing responses. “Single” includes both never-married and divorced participants.

**Table 2:** Prevalence of Physical and Neurocognitive Symptoms at Six Months Post-Infection (n = 212).

Symptom	Prevalence, n (%)
Physical Symptoms	
Severe fatigue after mild activity	49 (23.2%)
Chronic fatigue	48 (22.7%)
Headache	40 (19.0%)
High fever	28 (13.3%)
Dizziness	26 (12.3%)
Chronic pain	23 (10.9%)
Shortness of breath	18 (8.5%)
Sleep disturbance	16 (7.6%)
Abdominal pain	13 (6.2%)
Severe infection	12 (5.7%)
Neurocognitive Symptoms	
Difficulty retaining new information	122 (57.9%)
Difficulty concentrating	110 (52.1%)
Memory lapse	101 (47.9%)
Slowed thinking	73 (34.6%)
Difficulty making decisions	59 (28.0%)
Confusion	44 (20.9%)
Feeling disoriented	41 (19.4%)

**Table 3:** Associations Between Physical Symptoms and Cognitive Complaints Subdomains.

Physical Symptom	Prevalence, n (%)	Total Cognitive Complaints Mean (SD) t, *p*-Value	Memory Impairment Mean (SD) t, *p*-Value	Attention Impairment Mean (SD) t, *p*-Value	Cohen’s d
Severe Fatigue After Mild Activity	49 (23.2%)				0.46
No	162 (76.8%)	33.41 (8.09)	15.94 (4.21)	17.47 (5.20)	
Yes	49 (23.2%)	37.26 (9.00)	18.02 (4.53)	19.24 (5.64)	
*t*-test (t, df, *p*)		−2.84, 209, 0.005 *	−2.97, 209, 0.003 *	−2.05, 209, 0.041 *	
Chronic Fatigue	48 (22.7%)				-
No	163 (77.3%)	33.93 (8.50)	16.30 (4.35)	17.62 (5.32)	
Yes	48 (22.7%)	35.60 (8.23)	16.85 (4.44)	18.75 (5.42)	
*t*-test (t, df, *p*)		−1.21, 209, 0.227	−0.77, 209, 0.442	−1.28, 209, 0.201	
Headache	40 (19.0%)				0.36
No	171 (81.0%)	33.73 (8.31)	16.10 (4.31)	17.63 (5.22)	
Yes	40 (19.0%)	36.77 (8.72)	17.82 (4.38)	18.95 (5.78)	
*t*-test (t, df, *p*)		−2.07, 209, 0.040 *	−2.27, 209, 0.024 *	−1.41, 209, 0.161	
Dizziness	26 (12.3%)				-
No	185 (87.7%)	33.92 (8.30)	16.22 (4.37)	17.71 (5.25)	
Yes	26 (12.3%)	37.04 (9.16)	17.92 (4.26)	19.11 (5.95)	
*t*-test (t, df, *p*)		−1.77, 209, 0.078	−1.88, 209, 0.062	−1.26, 209, 0.210	
Chronic Pain	23 (10.9%)				-
No	188 (89.1%)	34.04 (8.37)	16.27 (4.34)	17.77 (5.24)	
Yes	23 (10.9%)	36.52 (8.93)	17.74 (4.43)	18.78 (6.22)	
*t*-test (t, df, *p*)		−1.33, 209, 0.184	−1.53, 209, 0.127	−0.86, 209, 0.393	
Shortness of Breath	18 (8.5%)				0.54
No	193 (91.5%)	33.92 (8.10)	16.30 (4.34)	17.62 (5.12)	
Yes	18 (8.5%)	38.44 (10.97)	17.78 (4.61)	20.67 (6.90)	
*t*-test (t, df, *p*)		−2.19, 209, 0.030 *	−1.37, 209, 0.171	−2.33, 209, 0.021 *	
Sleep Disturbance	16 (7.6%)				-
No	195 (92.4%)	34.06 (8.32)	16.31 (4.30)	17.75 (5.27)	
Yes	16 (7.6%)	37.31 (9.69)	17.87 (5.03)	19.43 (6.21)	
*t*-test (t, df, *p*)		−1.48, 209, 0.139	−1.38, 209, 0.168	−1.21, 209, 0.227	
Abdominal Pain	13 (6.2%)				-
No	198 (93.8%)	34.22 (8.14)	16.35 (4.28)	17.87 (5.11)	
Yes	13 (6.2%)	35.61 (12.61)	17.61 (5.63)	18.00 (8.42)	
*t*-test (t, df, *p*)		−0.57, 209, 0.566	−0.80, 12.92, 0.441	−0.05, 12.59, 0.958	

Notes: Prevalence is reported as n (% of 212). Means (SD) reflect total cognitive complaints, memory impairment, and attention impairment scores for participants reporting (Yes) and not reporting (No) each symptom. Scores were obtained from the Post-COVID Cognitive Impairment Scale (self-report); higher scores indicate greater self-reported impairment. One participant was excluded due to missing cognitive scores, resulting in an analysis sample of 211 (df = 209). *t*-tests compare scores between groups (t, df, *p*), with degrees of freedom adjusted where Levene’s test indicated unequal variances (e.g., abdominal pain). * *p* < 0.05 indicates statistical significance. Severe infection and high fever were excluded from this analysis as they were symptoms experienced during the acute infection phase, reported retrospectively to capture participants’ full symptom history, and are not considered long-term COVID-19 physical symptoms.

**Table 4:** Prevalence of Neurocognitive Symptoms and Associations with Demographics.

Neurocognitive Symptom	Prevalence, n (%)	Age (Mean Difference, *p*-Value)	Gender (Chi-Square, *p*-Value)	Education (Chi-Square, *p*-Value)
Difficulty retaining new information	122 (57.8)	−2.09, 0.151	4.51, 0.034 *	3.13, 0.537
Difficulty concentrating	110 (52.1)	2.73, 0.058	0.66, 0.613	22.38, 0.001 *
Memory lapse	101 (47.9)	1.59, 0.268	0.01, 0.907	5.17, 0.270
Slowed thinking	73 (34.6)	−0.40, 0.790	0.26, 0.455	2.75, 0.600
Difficulty making decisions	59 (28.0)	3.68, 0.021 *	7.03, 0.008 *	3.79, 0.435
Confusion	44 (20.9)	5.12, 0.001 *	1.99, 0.158	3.15, 0.533
Feeling disoriented	41 (19.4)	2.43, 0.183	0.01, 0.955	5.73, 0.221

Notes: Prevalence is n (% of 212). Age comparisons are independent-samples *t*-tests; values shown are mean difference (No − Yes) and p. * *p* < 0.05. Gender and education associations are chi-square tests (χ2(df) and p); df = 1 for gender and df = 4 for education. Detailed breakdowns and within-group percentages are provided in Table 5, Table 6 and Table 7.

**Table 8:** Education Level and Cognitive Complaint Severity at Six Months Post-Infection (n = 212).

Outcome	*F* (4, 207)	*p*	η^2^	Levene *p*	Significant Scheffé Contrasts (Higher Mean First)
Total cognitive complaints	4.70	0.001	0.083	0.177	Under Diploma > BA/BS (Δ*M* = 6.16, *p* = 0.043)
Memory impairment	3.25	0.013	0.059	0.610	Under Diploma > BA/BS (Δ*M* = 3.30, *p* = 0.035)
Attention impairment	4.64	0.001	0.083	0.120	Undergraduate > BA/BS (Δ*M* = 3.87, *p* = 0.016); Undergraduate > MA/MSC (Δ*M* = 3.78, *p* = 0.014)

Notes: Outcomes are continuous severity scores from the Post-COVID Cognitive Impairment Scale (higher = greater impairment). The Total Cognitive Complaints score represents the sum of the two primary subdomains—memory complaints and attention complaints—indicating the overall severity of self-reported cognitive difficulties. Homogeneity of variances held (Levene’s tests, ps shown), so standard one-way ANOVAs were used. Scheffé tests control family-wise error for all pairwise comparisons; only statistically significant contrasts are displayed. Group sizes (percent of total N = 212): Under Diploma n = 25 (11.8%), Undergraduate n = 35 (16.5%), BA/BS n = 60 (28.3%), MA/MSC n = 75 (35.4%), PhD n = 17 (8.0%).

**Table 9:** Infection History and Associations with Cognitive Complaints at Six Months Post-Infection (n = 212).

Infection Characteristic	Prevalence, n (%)	Total Cognitive Complaints Mean (SD)	Memory Impairment Mean (SD)	Attention Impairment Mean (SD)
Severe Infection (n = 211)				
No	199 (94.3%)	34.1 (8.6)	16.3 (4.4)	17.8 (5.4)
Yes	12 (5.7%)	37.1 (4.5)	18.3 (3.6)	18.8 (4.5)
*t*-test (t, df, *p*)		−2.06, 16.45, 0.055	−1.49, 209, 0.137	−0.63, 209, 0.537
High Fever (n = 211)				
No	183 (86.7%)	34.1 (8.2)	16.34 (5.2)	17.7 (5.2)
Yes	28 (13.3%)	36.0 (9.8)	17.00 (6.3)	19.0 (6.3)
*t*-test (t, df, *p*)		−1.14, 209, 0.256	−0.74, 209, 0.457	−1.19, 209, 0.236
Olfactory/Gustatory Dysfunction (n = 211)				
No	102 (48.3%)	33.7 (8.4)	15.8 (4.2)	17.9 (5.3)
Yes	109 (51.7%)	34.9 (8.5)	17.0 (4.4)	17.8 (5.5)
*t*-test (t, df, *p*)		−1.02, 209, 0.310	−2.11, 209, 0.036 *	0.10, 209, 0.916
Duration of Olfactory/Gustatory Dysfunction (days)	—	r = 0.029, *p* = 0.675	r = 0.092, *p* = 0.185	r = −0.028, *p* = 0.682
Number of COVID-19 Infections	—	r = 0.068, *p* = 0.324	r = 0.112, *p* = 0.106	r = 0.017, *p* = 0.811

Notes: Total sample N = 212. Prevalence values (second column) are reported as n (%) using the denominators indicated in parentheses after each infection characteristic (e.g., Severe infection, n = 211). For binary (dichotomous) predictors (severe infection, high fever, olfactory/gustatory dysfunction), group means (SD) are presented for the total cognitive complaints score, memory-impairment score, and attention-impairment score. The Total Cognitive Complaints score represents the sum of the two primary subdomains—memory complaints and attention complaints—indicating the overall severity of self-reported cognitive difficulties. Independent-samples *t*-tests compare participants with vs. without the characteristic (t, df, *p*); Welch’s correction was applied where Levene’s test indicated unequal variances. For continuous predictors, Pearson correlation coefficients (r) and two-tailed *p*-values are shown. Denominators differ from the total sample where missing responses occurred: n = 211 for severe infection, high fever, olfactory/gustatory dysfunction, and number of infections (one missing); n = 210 for duration of dysfunction (two missing). * *p* < 0.05.

**Table 10:** Multiple linear regression predicting total cognitive complaints in individuals with Long COVID.

Predictor	B	β	SE	t	*p*-Value
Constant	35.1	—	2.8	12.5	<0.0
Gender	0.3	0.0	1.2	0.3	0.8
Age	−0.0	−0.1	0.1	−0.7	0.5
Education	−1.2	−0.2	0.5	−2.59	0.0
Number of Physical Symptoms	0.7	0.2	0.3	2.68	0.0
Duration of olfactory/gustatory Dysfunction	−0.0	−0.0	0.0	−0.14	0.9
Number of Infections	0.2	0.0	0.8	0.27	0.8
		**R^2^**: 0.07		**Adjusted R^2^**: 0.04	

Note. Results of multiple linear regression including gender, age, education, number of physical symptoms, duration of olfactory/gustatory dysfunction, and number of infections as predictors of total cognitive complaints. Significant predictors are highlighted in bold. B = unstandardized coefficient; β = standardized coefficient; SE = standard error.

## Data Availability

The original data presented in the study are openly available in OSF (Open Science Framework) at: https://osf.io/85rxq/overview?view_only=ee8e941ba2c24f31b2c6fe0efebfc4f2 (accessed on 10 October 2025).

## Appendix A

**Table A1:** Reliability analysis of the Physical and Neurocognitive Symptom Checklist (pilot sample, n = 30).

Subscale	Example Items	Number of Items	Cronbach’s α
Physical symptoms	shortness of breath, severe infection, high fever, abdominal pain, headache, dizziness, chronic pain, chronic fatigue, headache, sleep disturbance, muscle weakness, sleep problems, severe fatigue after mild activity	10	0.83
Neurocognitive symptoms	difficulty concentrating, slowed thinking, confusion, memory lapses, feeling disoriented, difficulty making decisions, difficulty retaining new information	7	0.86
Total scale	—	17	0.85
